# Fabrication and sensing behavior of one-dimensional ZnO-Zn_2_GeO_4_ heterostructures

**DOI:** 10.1186/1556-276X-9-344

**Published:** 2014-07-09

**Authors:** Yuan-Chang Liang, Tzu-Yin Lin

**Affiliations:** 1 Institute of Materials Engineering, National Taiwan Ocean University, Keelung 20224, Taiwan

**Keywords:** Sensing properties, One-dimensional, ZnO-Zn_2_GeO_4_, Solid-state reaction, Heterostructure

## Abstract

Well-crystalline one-dimensional ZnO-Zn_2_GeO_4_ (ZGO) heterostructures were successfully synthesized using a high-temperature solid-state reaction between the ZnO and Ge layers of ZnO-Ge core-shell nanostructures. The polycrystalline ZGO crystallites had a thickness in the range of 17 to 26 nm. The high-temperature solid-state reaction induced grooves and crystal defects on the surfaces of the ZGO crystallites. The sensors made from the ZnO-ZGO heterostructures exhibited a marked photocurrent response to UV light at room temperature and a gas sensing response to acetone gas at 325°C. The observed sensing properties are attributed to the rugged surface of the ZGO heterointerfaces between ZnO and ZGO, surface crystal defects of ZGO, and cross-linked contact regions of ZnO-ZGO.

## Background

Binary wide-bandgap oxides are promising materials for optoelectronic, catalyst, and sensor applications [1,2]. To satisfy the different requirements of device applications, binary oxides doped with various dopants were studied to improve the intrinsic characteristics and increase the functionality of the oxides [3-5]. Binary oxides with a one-dimensional (1D) morphology show particular potential for nanodevice applications because of their high surface-to-volume ratios. Among various binary oxides, 1D ZnO is one of the most commonly used materials for nanodevices because the quality of its synthetic processes is satisfactory [4,6].

In addition to controlling the composition of binary oxides by doping, construction of an oxide heterostructure enhances their functionality [7]. Several proposed ZnO-based binary heterostructures exhibit satisfactory physical and chemical properties. The one-step or two-step processes involving chemical solutions and/or thermal evaporation methodologies have been adopted for fabricating binary oxide heterostructures [8,9]. However, research on an oxide heterostructure consisting of a ternary oxide is still lacking. This is because synthesis of an oxide heterostructure with a 1D ternary oxide counterpart is technologically challengeable [10-12]. A high-temperature solid-state reaction is a feasible methodology to form a ternary oxide by using constituent binary oxides [11,12]. A small ionic radius difference between Ge and Zn ions increases the probability of the Ge ion replacing the Zn ion. Incorporating Ge into a ZnO crystal changes the optical properties of ZnO through modification of the electronic structure around the band edge [13]. Moreover, Zn_2_GeO_4_ (ZGO) is a ternary wide-bandgap semiconductor and a native defect phosphor exhibiting white luminescence under UV light excitation [14]. Lin et al. showed that hydrothermally synthesized ZGO rods annealed at 1,000°C exhibit satisfactory photocatalytic hydrogen generation [15]. Solvothermally synthesized ZGO nanostructures have been studied for the photocatalytic reduction of CO_2_ to CH_4_[16]. In addition to photocatalytic applications, research on structure-dependent sensing characteristics of a single 1D ZGO or ZnO-ZGO heterostructure has been limited [17]. In this study, a 1D ZnO-ZGO heterostructure was synthesized using a high-temperature solid-state reaction of ZnO-Ge core-shell nanostructures. The correlation between the structural properties and potential application of such structures in UV photodetectors and gas sensors was investigated.

## Methods

Cross-linked ZnO nanostructures were used as the substrate for the growth of Ge nanofilms onto ZnO nanostructures to form ZnO-Ge core-shell nanostructures. The experimental setup for the preparation of cross-linked ZnO nanostructures has been published elsewhere [12]. Deposition of Ge nanofilms was performed using a radio-frequency magnetron-sputtering system. During deposition, the substrate temperature was maintained at room temperature and the deposition gas pressure was fixed at 20 mTorr, with pure Ar ambient. The as-synthesized ZnO-Ge samples were further annealed in air at 800°C for 30 min to form ZnO-ZGO heterostructures.

Crystal structures of the samples were investigated by X-ray diffraction (XRD) using Cu Kα radiation. X-ray photoelectron spectroscopy (XPS) analysis was used to determine the chemical binding states of the constituent elements. The morphologies of the as-synthesized samples were characterized by scanning electron microscopy (SEM), and high-resolution transmission electron microscopy (HRTEM) was used to investigate the detailed microstructures of the samples. Room temperature-dependent photoluminescence (PL) spectra were obtained using the 325-nm line of a He-Cd laser. The UV photoresponse of the samples was measured at a fixed external voltage of 5 V with and without UV irradiation. To measure gas sensing properties, heterostructure samples were placed in a closed vacuum chamber and various concentrations of acetone gas were introduced into the chamber, using dry air as the carrier gas. Silver glues were laid on the surfaces of the samples to form two contact electrodes, and the samples were fixed at 325°C during gas sensing test. Sensor response to test gases was defined as *I*_g_/*I*_a_, where *I*_a_ is the current in air and *I*_g_ is the current in the test gas.

## Results and discussion

**Figure 1:**
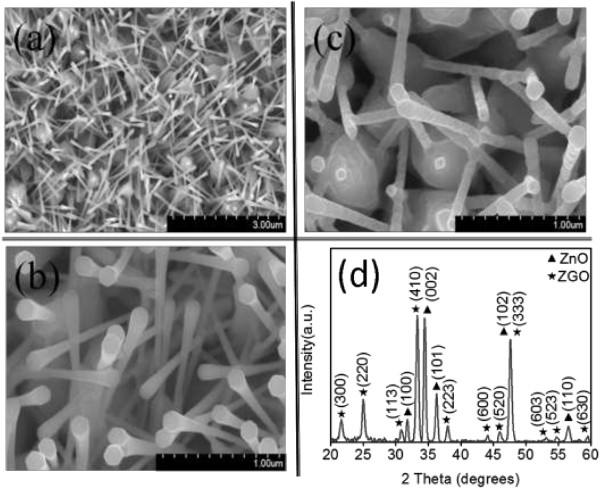
**SEM images of ZnO and ZnO-Ge nanostructures and SEM image and XRD pattern of ZnO-ZGO heterostructures. (a)** Low-magnification SEM image of the ZnO nanostructures. **(b)** High-magnification SEM image of the ZnO-Ge nanostructures. **(c)** High-magnification SEM image of the ZnO-ZGO heterostructures. **(d)** XRD pattern of the ZnO-ZGO heterostructures.

Figure 1a shows a low-magnification SEM micrograph of the as-synthesized ZnO structures, which comprised two features. The lower part of the ZnO structure exhibited a coarse rodlike feature, whereas the upper part of the structure was relatively thin in diameter and had a hexagonal cross-sectional morphology. The diameter of the upper part of the structure in Figure 1a was approximately 70 to 130 nm, and the surfaces of the as-synthesized samples were smooth. No marked change in the morphology of the as-synthesized sample occurred after deposition with a thin Ge layer (ZnO-Ge nanostructures) by sputtering (Figure 1b). In contrast, the morphology of the ZnO-Ge nanostructures, after high-temperature annealing at 800°C, developed irregular and rough features (Figure 1c). This indicated that a solid-state reaction between the ZnO core and Ge shell materials occurred at such a high annealing temperature [12,18]. Figure 1d shows an XRD pattern for the ZnO-Ge nanostructures annealed at 800°C. Several intense ZnO Bragg reflections were observed, which we assigned to the (100), (002), (101), (102), and (110) planes. The XRD spectrum indicated multiple crystallographic orientations of the ZnO crystals, which is consistent with the randomly cross-linked ZnO morphology observed in the SEM micrograph. Moreover, several clear Bragg reflections of the ZGO phase exhibiting a rhombohedral crystal structure were present in the XRD spectrum (JCPDS No. 11-0687). The XRD spectrum showed well-crystalline ZGO crystals covering the cross-linked ZnO nanostructures. The thermal annealing condition in the current study successfully induced the outer Ge thin layer to solid-state react with inner ZnO crystallites to form ternary ZGO crystallites.

**Figure 2:**
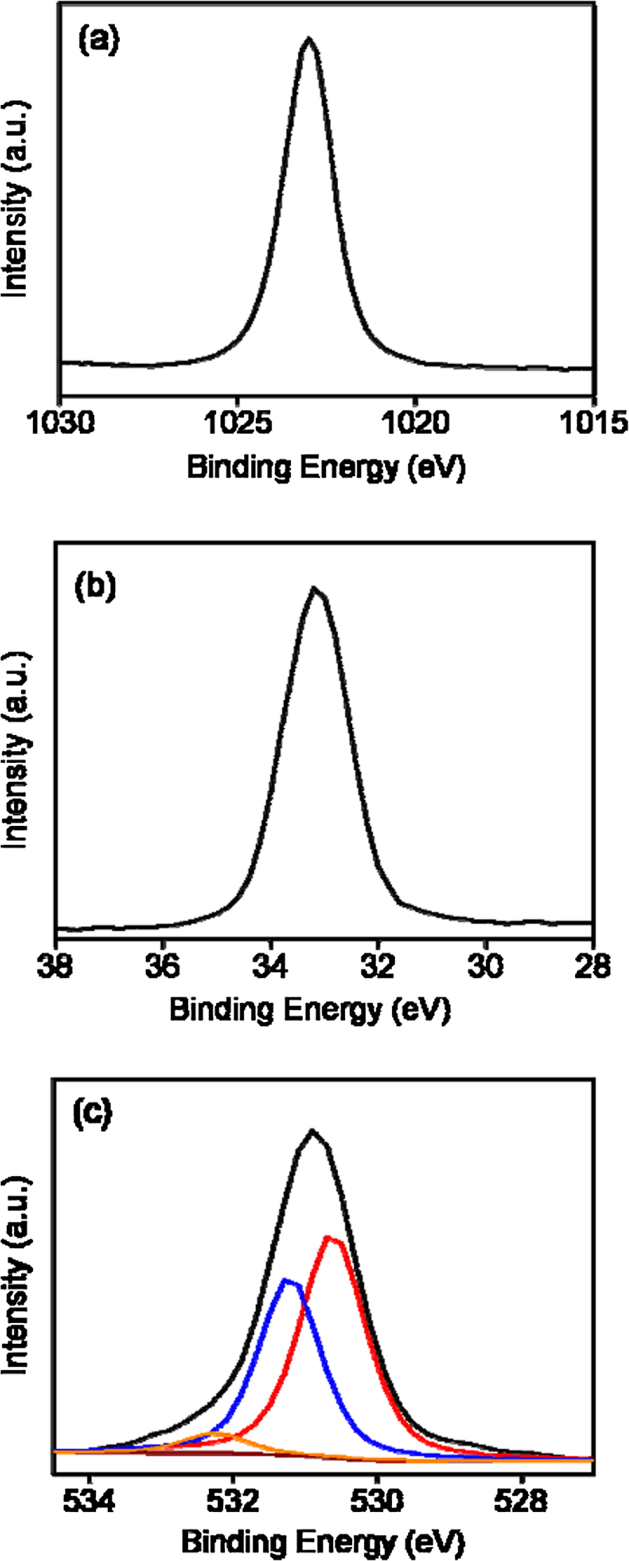
**XPS narrow-scan spectra from the ZGO crystallites. (a)** XPS narrow-scan spectrum of Zn 2p_3/2_. **(b)** XPS narrow-scan spectrum of Ge 3d. **(c)** XPS narrow-scan spectrum of O 1 s.

Figure 2 presents the narrow-scan spectra of ZnO-ZGO for the elements Zn, Ge, and O. Figure 2a shows that the Zn 2p_3/2_ peak was centered at approximately 1,022.4 eV. This value is consistent with the reported binding energy for Zn^2+^ in the bulk zinc oxide [12]. Figure 2b shows that the main Ge 3d peak position was located at 33.1 eV. This binding energy corresponds to the Ge^4+^ coordination site on the GeO_2_ surface [19,20]. Figure 2c illustrates an asymmetric O 1 s peak of the sample. The O 1 s peak can be resolved into three components. The lower binding energy component arises from oxygen in the oxide. The middle binding energy component may represent oxygen ions in the oxygen-deficient regions within the oxide matrix. The formation of oxygen vacancy defects might be associated with a phase transformation of the sample during a high-temperature solid-state reaction. The highest binding energy (532.3 eV) indicates the presence of hydroxyl groups on the sample surfaces resulting from oxygen vacancies on the surfaces of the sample with a high surface-to-volume ratio [6,21].

**Figure 3:**
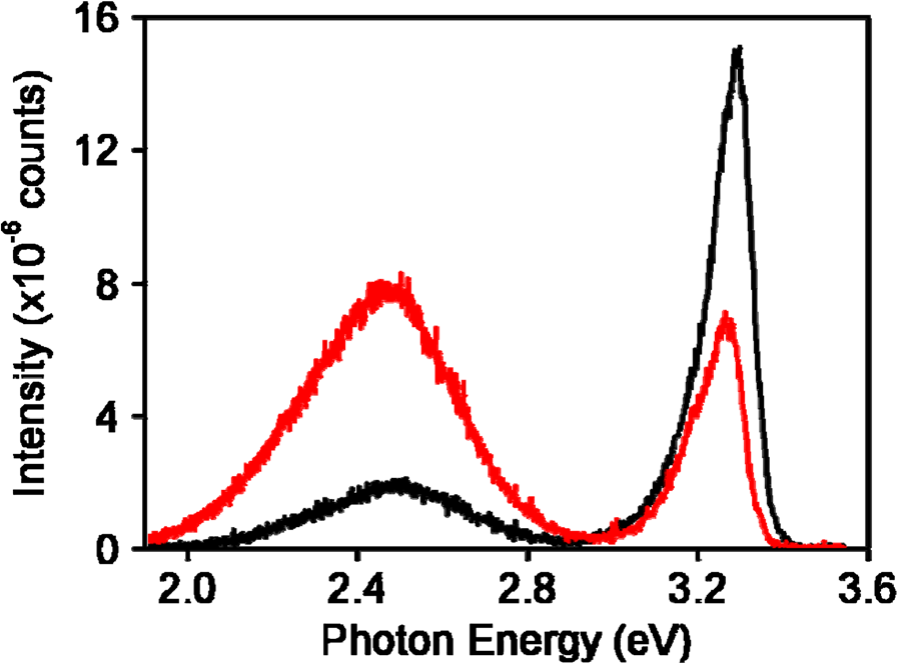
PL spectra of the ZnO-Ge (black line) and ZnO-ZGO (red line) heterostructures.

The PL spectrum for ZnO-ZGO was measured; moreover, the PL spectrum for ZnO-Ge was compared to understand the luminescence properties of ZnO-ZGO (Figure 3). A distinct UV light emission band was present at approximately 3.3 eV, which we ascribed to the near-band edge emission of ZnO [6,22]. Moreover, a clear visible light emission band was present at approximately 2.5 eV for ZnO-Ge and ZnO-ZGO. Comparatively, the UV light emission band intensity was quenched, and the visible light emission band intensity was markedly enhanced when ZnO-Ge transformed into ZnO-ZGO after a high-temperature annealing process. The peak position of the visible light emission band is similar to those of previous studies of nanostructured ZGO phosphors [23]. The visible light emission band for ZGO originates from its native defects [24]. The formation of the ternary ZGO compound through a high-temperature solid-state reaction might involve the formation of native defects, such as oxygen vacancies, in the ZGO crystals [18]. This is supported by our XPS O 1 s analysis, which indicated oxygen vacancies in the ZGO lattice. The solid-state reaction induced crystal defects in ZnO-ZGO, which might account for the difference in the PL spectra between ZnO-Ge and ZnO-ZGO.

**Figure 4:**
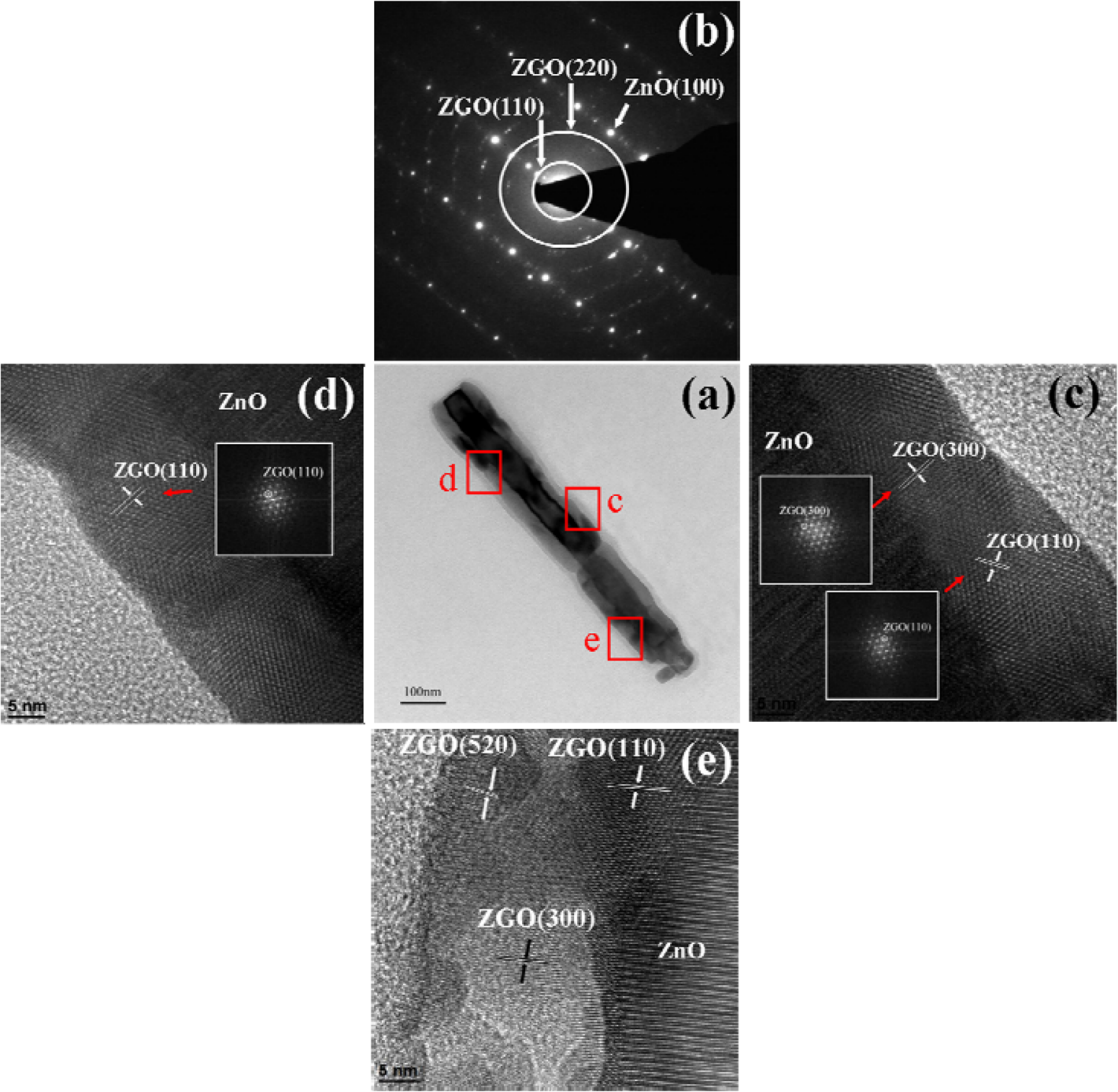
**Low- and high-magnification TEM images and electron diffraction pattern of the ZnO-ZGO heterostructure. (a)** Low-magnification TEM image of a single ZnO-ZGO heterostructure. **(b)** Electron diffraction pattern of the heterostructure. **(c, d, e)** High-resolution images of the heterostructure taken from various regions. The corresponding FFT images taken from the local lattice fringes are also shown in the insets.

Figure 4a presents a TEM image of the morphology of a single 1D ZnO-ZGO heterostructure, showing that the surface of ZnO-ZGO was rugged. Figure 4b shows the electron diffraction pattern of the ZnO-ZGO structure. Tiny spots formed clear ringlike patterns associated with polycrystalline ZGO crystals. Moreover, sharp, bright, large spots appeared to emanate from the ZnO layer of the ZnO-ZGO structure. Figure 4c,d,e shows high-resolution images of various regions of the ZnO-ZGO structure. In Figure 4c,d, small surface groves are present on the structure. Clear, ordered lattice fringes present on the outer layer of the structure are assigned to the ZGO crystalline phase according to the fast Fourier transform pattern (insets in Figure 4c,d). The interplanar *d*-spacing evaluated based on the lattice fringes was approximately 0.71 nm, which corresponds to the {110} lattice planes of the well-crystalline ZGO structure. The corresponding 0.41 nm is ascribed to the {300} lattice planes. Moreover, Figure 4e shows that the arrangement of lattice fringes of the ZGO layer is relatively more random than that in Figure 4c,d. The multiple {110}-, {300}-, and {520}-oriented lattice fringes are presented in Figure 4e. The HRTEM image analysis results indicated that some ZGO crystallites formed a favorable crystallographic match with the ZnO crystal, whereas others showed multi-oriented features. According to the TEM observation, the thickness of the ZGO crystallites ranged from approximately 17 to 26 nm.

**Figure 5:**
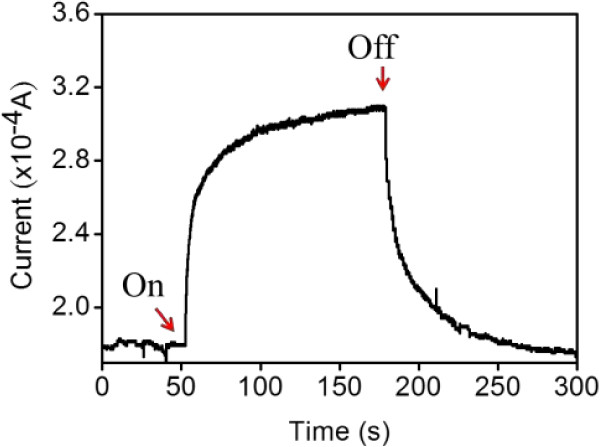
Time-dependent current variation of the ZnO-ZGO heterostructures measured in air ambient with and without UV light irradiation.

Figure 5 shows the dynamic UV light photoresponse curve of ZnO-ZGO measured in ambient air at room temperature. ZnO-ZGO shows UV light photocurrent sensitivity. The increase and decrease of photocurrents show a time-dependent function in the presence and absence of UV lights, respectively. The dark current for ZnO-ZGO was approximately 1.8 × 10^-4^ A, and the UV-irradiated current was approximately 3.1 × 10^-4^ A. The corresponding resistance variation of the sample was large. The resistance of the sample was approximately 27 kΩ for the UV-off state and 16 kΩ for the UV-on state. A difference of approximately 11 kΩ existed in the sample with and without UV irradiation. Such a high resistance difference guarantees an efficient UV light photoresponse for ZnO-ZGO. A UV light photoresponse phenomenon has been observed in other semiconductor systems with an explanation of Schottky barrier models [25]. The photoconductive gain of the nanostructures was posited with the presence of oxygen-related hole-trap states at the nanostructure surface [26]. Previous research has indicated that the photoresponse of a nanostructure-based photodetector is highly surface-size-dependent [27]. The observed photoresponse property of ZnO-ZGO is attributed to the rugged surface and oxygen vacancy in the ZGO crystallites. These factors increase the adsorption of oxygen and water molecules; thus, an efficient UV light photoresponse was obtained for ZnO-ZGO. The response time and recovery time for the photodetector were defined as the time for a 90% change to occur in photocurrents upon exposure to UV light and to the UV-off state in the current study. The response time was approximately 44 s and the recovery time was 25 s. The response time of ZnO-ZGO in the UV-on state was considerably longer than that in the UV-off state. This indicates that charge separation during UV light irradiation dominates the efficiency of the photodetector composed of ZnO-ZGO [18].

**Figure 6:**
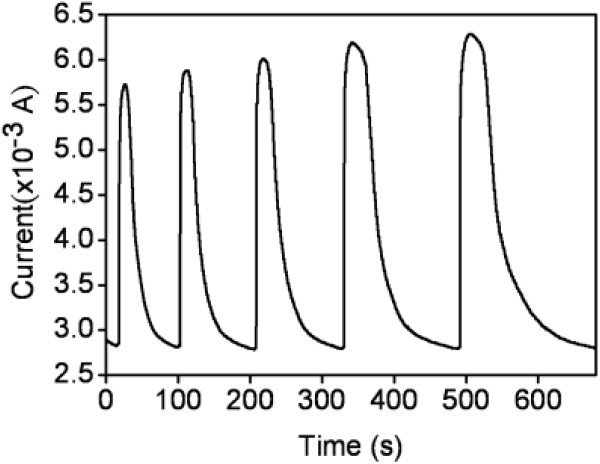
Time-dependent current variation of the ZnO-ZGO heterostructures upon exposure to various acetone concentrations (50, 100, 250, 500, and 750 ppm) at 325°C.

Figure 6 shows the dynamic gas sensor responses (currents vs. time) of the ZnO-ZGO sensor to acetone gas. The ZnO-ZGO sensor was tested at operating temperatures of 325°C with acetone concentrations of 50 to 750 ppm. The current of the sample increased upon exposure to acetone and returned to the initial state upon the removal of the test gas. The changes in gas sensor response (*I*_g_/*I*_a_) for the sample showed a clear dependence on acetone concentration. The gas sensor response increased with acetone concentration. The response of the ZnO-ZGO sensor to 50 ppm acetone was 2.0, and that to 750 ppm acetone was approximately 2.4. We further evaluated the gas response and recovery speeds of the ZnO-ZGO sensor. The response time and recovery time were defined as the time for a 90% change in current to occur upon exposure to acetone and to air, respectively. The response time for the ZnO-ZGO sensor increased from 5.3 to 5.7 s when the acetone concentration was increased from 50 to 750 ppm, respectively. No substantial difference in response time was observed when the sensor was exposed to various acetone concentrations (50 to 750 ppm). However, a marked difference in recovery time was observed for the sensor upon exposure to various acetone concentrations. The recovery time increased from 21 to 89 s when the acetone concentration was increased from 50 to 750 ppm. Comparatively, the response time was shorter than the recovery time for the gas sensor in this study. The gas sensing mechanism for n-type semiconductor oxide sensors is surface-controlled and is controlled by the species and amount of oxygen ions on the surface [28]. The difference between the response time and recovery time revealed that the desorption reaction of oxygen molecules (release of electrons) was faster than the adsorption process of oxygen molecules (trapping of electrons) on the surface of the sample. A similar phenomenon was observed in a ZnO-based sensor tested in a reduced-gas environment [29]. Because the thickness of the ZGO crystallites ranges from 17 to 26 nm, the variation in resistance for the ZnO-ZGO sensor during gas sensing tests might be determined according to the resistance of the ZGO crystallites and contact regions between each cross-linked structure. Contact between oxides results in the formation of potential barriers [30,31]. Recently, cross-linked 1D oxide nanostructures have indicated that potential barriers formed at the contact regions play a crucial role in affecting gas sensing performance [32]. Efficient ethanol gas sensing for n-type 1D oxide nanostructures is attributed to electron donor-related oxygen vacancies in the nanostructures [33]. These factors induced numerous depletion regions in ZnO-ZGO when exposed to ambient air in the current study; a clear resistance variation was further achieved in the sample upon exposure to the acetone gas.

## Conclusions

We successfully prepared ZnO-ZGO heterostructures for UV light photoresponse and acetone gas sensing applications by the sputter deposition of Ge ultrathin films onto ZnO nanowire templates after a high-temperature solid-state reaction. The ZGO crystallites were homogeneously formed on the surface of the residual ZnO underlayer, exhibiting a rugged morphology. The XPS spectra and PL spectrum of the ZnO-ZGO heterostructures indicated the existence of surface crystal defects. The ZnO-ZGO heterostructures exhibited clear photocurrent sensitivity to UV light at room temperature and a gas sensing response to acetone in a concentration range of 50 to 750 ppm at 325°C. The detailed structural analyses in this study accounted for the observed UV light photoresponse and acetone gas sensing properties of the ZnO-ZGO heterostructures.

## Competing interests

The authors declare that they have no competing interests.

## Authors’ contributions

YCL designed the experiments and drafted the manuscript. TYL carried out the sample preparations and the material analyses. Both authors read and approved the final manuscript.

## Authors’ information

YCL is a professor of the Institute of Materials Engineering at National Taiwan Ocean University (Taiwan). TYL is a graduate student of the Institute of Materials Engineering at National Taiwan Ocean University (Taiwan).
